# Psoriatic arthritis and COVID-19: a new challenge for rheumatologists and dermatologists

**DOI:** 10.1186/s12969-023-00929-1

**Published:** 2024-01-18

**Authors:** Zohreh Jadali

**Affiliations:** Department of Immunology, School of Public Health, Tehran University of Medical Sciences, 6446, Tehran, 14155 Iran

**Keywords:** SARS-CoV-2, COVID-19, Psoriatic arthritis

## Abstract

COVID-19 has changed the global health system and has great impact on different types of medical specialties including, dermatology and rheumatology. This point is important because although these two specialties are distinct subfields of medicine, there is some overlap between them. The overlap can be described by a number of rheumatic diseases that have cutaneous manifestations and vice versa. A good example of this is psoriatic arthritis because, in up to 42% of people, cutaneous lesions and arthritis coexist. Interestingly, emerging reports have described the possible occurrence of psoriasis and psoriatic arthritis in COVID-19 patients. Although the exact mechanism is unclear, some common pathophysiological mechanisms may contribute to disease pathogenesis. Therefore, elucidation of shared pathological pathways that connect these diseases will be valuable for better diagnosis and the complete treatment of COVID-19 patients with cutaneous and rheumatologic diseases.

## Dear Editor

There is now growing evidence of COVID-19-triggered rheumatic and skin disorders [1]. Some of them, like psoriatic arthritis(PsA), have combined cutaneous and joint lesions [2].

The co-occurrence of PsA and COVID-19 can complicate diagnosis and treatment. Therefore, exploration of the shared molecular mechanisms between COVID-19 and PsA may explain some causes of cutaneous and rheumatologic symptoms that occur in COVID-19 patients.

Both COVID-19 and PsA pose a challenge to the body’s immune system. Thus, an important question is how common molecular mechanisms may link the immune system with PsA and COVID-19. Evidence suggests that angiotensin*-*converting enzyme 2(ACE2) is a key molecule connecting **t**hese two conditions because there is a correlation between tissue-specific expression of ACE2(a primary receptor for virus entry) and COVID-19 susceptibility. Therefore, ACE2 expression in human tissues that can be affected during PsA may increase the risk of PsA in infected patients.

The ACE2 expression in human synovial tissues, the basal epidermal layer, or keratinocytes, indicates a new potential skin and joint injury mechanism among patients. The binding of severe acute respiratory syndrome coronavirus 2(SARS-CoV-2) spike protein to ACE2 may initiate the pathogenic cascade of skin and rheumatic diseases in infected patients.

An association of higher ACE2 expression with COVID-19 adverse effects and elevated expression of ACE2 in psoriatic lesional skin or active rheumatoid synovium may also increase tissue susceptibility to disease progression. Virus presence in the skin or synovial fluid of patients raises the possibility that SARS-CoV‐2 can be a pathologic stimulus for synovial or skin inflammation [3].

Transmission of SARS-CoV-2 to skin and joints may also occur indirectly, through interaction with ACE2 that is expressed on the surface of lymphatic endothelial cells or the cutaneous vasculature. Virus-endothelial interaction can result in the recruitment of inflammatory cells and initiation of the inflammation cascade.

An overactive immune system and dysregulated inflammatory responses, after COVID-19 seem to play a role in skin and joint damage. This process can result from different mechanisms, including molecular mimicry, epitope spreading, or bystander activation [4].

Drug side effects are another important aspect because certain medications which were initially believed promising agents for COVID-19 treatments are the known causes of drug-induced psoriasis. This is supported by findings including the exacerbation of PsA in COVID-19 patients who were treated with hydroxychloroquine [5].

Overall, a possible relationship may exist between COVID-19 and PsA. So, clarification of the disease mechanisms is necessary to get an accurate diagnosis and effective treatment.

## Data Availability

Not applicable.

## Abbreviations

SARS-CoV-2: Severe acute respiratory syndrome coronavirus 2
COVID-19: Coronavirus disease 2019
ACE2: Angiotensin-converting enzyme 2
PsA: Psoriatic arthritis
